# Regulation of Cisplatin Resistance in Lung Cancer Cells by Nicotine, BDNF, and a β-Adrenergic Receptor Blocker

**DOI:** 10.3390/ijms232112829

**Published:** 2022-10-24

**Authors:** Ravel Ray, Hind Al Khashali, Ben Haddad, Jadziah Wareham, Kai-Ling Coleman, Danyah Alomari, Robert Ranzenberger, Jeffrey Guthrie, Deborah Heyl, Hedeel Guy Evans

**Affiliations:** Chemistry Department, Eastern Michigan University, Ypsilanti, MI 48197, USA

**Keywords:** β-adrenergic receptors, brain-derived neurotrophic factor, lung cancer, cisplatin, nicotine, EGFR, IGF-1R, PI3K/AKT, chemoresistance

## Abstract

It is well-recognized that cigarette smoking is a primary risk factor in the development of non-small cell lung cancer (NSCLC), known to account for ~80% of all lung cancers with nicotine recognized as the major addictive component. In investigating the effect of nicotine, brain-derived neurotrophic factor (BDNF), and the β-adrenergic receptor blocker, propranolol, on sensitivity of NSCLC cell lines, A549 and H1299, to cisplatin, we found increased cell viability, and enhanced cisplatin resistance with nicotine and/or BDNF treatment while opposite effects were found upon treatment with propranolol. Cell treatment with epinephrine or nicotine led to EGFR and IGF-1R activation, effects opposite to those found with propranolol. Blocking EGFR and IGF-1R activation increased cell sensitivity to cisplatin in both cell lines. PI3K and AKT activities were upregulated by nicotine or BDNF and downregulated by cell treatment with inhibitors against EGFR and IGF-1R and by propranolol. Apoptosis and cell sensitivity to cisplatin increased upon co-treatment of cells with cisplatin and inhibitors against PI3K or AKT. Our findings shed light on an interplay between nicotine, BDNF, and β-Adrenergic receptor signaling in regulating survival of lung cancer cells and chemoresistance which can likely expand therapeutic opportunities that target this regulatory network in the future.

## 1. Introduction

Compared to other cancer types, lung cancer remains the leading cause of cancer-related deaths worldwide with smoking representing the major risk factor for non-small cell lung cancer (NSCLC) and small cell lung cancer [1].

Cisplatin is a small molecule platinum compound that is part of the platinum-based antineoplastic drugs [2,3]. Interaction of cisplatin with DNA is the root cause of its cytotoxic effects, generating covalent DNA–platinum adducts, leading to induction of a DNA damage response in tumor cells [4]. As a chemotherapeutic drug, cisplatin induces genotoxic stress via broad spectrum clinical activity against a variety of tumors, especially lung cancers, which can contribute to apoptosis or chemoresistance [2,5]. For decades, cisplatin has been used for the treatment of patients with numerous types of cancer, including lung, inducing tumor death via activation of proapoptotic mechanisms [3]. Cisplatin sensitivity was reported to increase in NSCLC cells upon inhibition of eukaryotic translation initiation factor 5A2 and regulation of the epithelial–mesenchymal transition [4]. Cisplatin combinations are currently the most effective therapy for advanced NSCLC; however, despite an initial positive reaction to cisplatin-based chemotherapy, cisplatin resistance, relapses, and treatment failures eventually occur [3]. 

Patients who smoke have been widely reported to have a poor response to cancer chemotherapy compared to non-smokers [6,7]. Strong association has been documented between NSCLC and smoking, considered to be the greatest, and most preventable, risk factor for developing lung cancer [8,9,10]. While nicotine, the major addictive component of tobacco smoke, is not considered carcinogenic and is unable to promote tumorigenesis in humans, it is able to activate mitogenic signaling pathways increasing cell proliferation, growth, survival, and metastasis [6,11,12]. It also enhances chemoresistance of cancer cells by blocking apoptosis induced by certain chemotherapeutic agents, including cisplatin, used for the treatment of NSCLCs [10,13,14]. Locally advanced NSCLC cells are known to be highly resistant to chemoradiotherapy [4,15,16]. Resistance to antineoplastic drugs-induced apoptosis, along with poor prognosis, are characteristics of NSCLC [4,9,10,17]. 

Acetylcholine (ACh) is traditionally known as a neurotransmitter that mediates synaptic transmission. However, ACh and cholinergic proteins have also been detected in non-neuronal tissues, including lung [18,19,20,21]. ACh plays an important role in lung homeostasis and functions as an autocrine and paracrine growth factor in human lung cancer cells known to express the proteins necessary for synthesis and degradation of ACh [18]. ACh binds to nicotinic acetylcholine receptors (nAChRs) which are members of the pentameric ligand-gated ion channels, and muscarinic receptors on lung cancer cells accelerating their proliferation [18,21,22,23,24]. Accumulating evidence has shown that nAChRs play a crucial role in cholinergic signaling and in the initiation, growth, survival, proliferation, progression, and metastasis of a variety of cancers including lung cancer [6,7,11,18,22,25]. The nAChRs bind ACh and nicotine as the endogenous and exogenous ligand, respectively [6,18,26]. Nicotine is widely recognized to accelerate growth and metastasis of lung cancers mediated via nAChRs on lung tumors that normally bind the endogenous ligand, ACh [18,19,22]. Treatment with nicotine was found to elevate the levels of ACh in human lung cancer cells, including A549 and H1299 cells, thereby, increasing their proliferation [18,27]. The nAChRs have been detected in human NSCLC cell lines that include A549 and H1299, which were used in this study [19,28]. Exposure to nicotine by smoking or by nicotine supplements can activate nAChRs, promoting NSCLC growth and progression, and lung metastasis [7]. While ACh is the physiological ligand for nAChRs, nicotine can displace Ach, since it binds these receptors with higher affinity than ACh [6,7,11,22,25,29]. 

In numerous non-neuronal cells, multiple nAChR subunits are known to be expressed. However, the main receptor facilitating nicotine-mediated cell growth is thought to be the homomeric α7-nAChR [6,7,22,25]. Previous reports have demonstrated that stimulation with nicotine could induce proliferation in a variety of in vitro cell culture models, an effect that could be abrogated by nAChR antagonists, such as the α7 antagonist α-bungarotoxin (α-BTX), which blocks the proliferative effects of nicotine, pointing to α7-nAChR as a potential target for cancer therapy [22,30]. Using lung cancer mouse models, similar effects have also been shown in vivo, due to nicotine that led to increased tumorigenesis and metastasis [7,12]. 

Lung cancer cells have been shown to express β-adrenergic receptors (β-ARs), a class of seven-transmembrane G protein-coupled receptors (GPCRs), that function to facilitate cellular proliferation, apoptosis resistance, and metastasis [31]. In addition to binding of nicotine to nAChRs, that act as the principal sites for its biological action, nicotine signaling has also been shown to activate β-Ars, triggering the production of β-AR ligands, for example, adrenaline and noradrenaline, which contribute to cell proliferation and lung cancer development [7,8]. Nicotine has also been shown to bind the β-ARs activating a number of mitogenic and oncogenic signaling pathways [8]. 

Several reports suggest that stimulation of β-ARs leads to cellular proliferation, tumorigenesis, and chemotherapeutic resistance [31,32]. Treatment of NSCLC A549 cells with cisplatin and the non-selective β-blocker, propranolol, that competitively antagonizes β-ARs, was found to have antitumor activity, decreased cell survival, increased apoptosis, and enhanced sensitivity of A549 cells to cisplatin in vitro, thus, suggesting that propranolol acts as a therapeutic sensitizer [32].

Brain-derived neurotrophic factor (BDNF) is a member of the neurotrophin family of growth factors, that along with its high affinity primary receptor, tropomyosin receptor kinase B (TrkB), is widely recognized for its role in the survival of neurons and synapses [33]. Many studies have, more recently, highlighted the emerging role of neurotrophins in different cancers [33,34,35]. BDNF was reported to be involved in tumor pathogenesis, exerting carcinogenic effects in different types of cancer [36,37], including lung, promoting proliferation and invasion of lung squamous cell carcinoma [36,37,38]. Compared with normal lung, BDNF levels were upregulated in lung cancer cell culture supernatants, linked to lung tumorigenesis, and associated with poor prognosis in NSCLC patients [36]. TrkB was reported to play a critical role in lung cancer development and its deficiency induced apoptosis and significantly inhibited metastasis of a lung adenocarcinoma model [36]. Silencing BDNF expression was shown to block cell proliferation, promoting cell apoptosis, and thereby blocking the growth of lung cancer cells [36,37,38,39]. We recently explored the relative abundance of proBDNF and mature BDNF (mBDNF) in A549 (p53 wild-type) and H1299 (p53-null) lung cancer cell media and reported higher levels of proBDNF in the media of A549 cells than in H1299 cell media [40]. Treatment with BDNF attenuated cisplatin-induced cell death and decreased the chemosensitivity of neuroblastoma cells to cisplatin, an effect that was blocked by cell treatment with the PI3K inhibitor, LY294002, suggesting a role of PI3K in BDNF’s rescue effects [41]. Our laboratory recently showed that treatment with acetylcholinesterase, well-known for its classical role in the catalytic hydrolysis of cholinergic neurotransmitters, shifted processing of the amyloid precursor protein away from the non-amyloidogenic pathway and toward the amyloidogenic pathway, whereas treatment with BDNF led to opposite effects [42]. While nicotine was earlier reported to regulate changes in the levels of BDNF that can affect nAChRs, and vice versa, in the brain from animal studies, relatively little is known about the interplay between nicotine and BDNF in NSCLC [38,39,43,44].

This study examines parallel and/or converging contributions of neurotrophic, neurotransmitter, and growth factor receptors in regulating chemoresistance in NSCLC. Specifically, the study focuses on how nAChR, TrkB, and β-AR, lead to regulation of EGFR and IGF-1R in NSCLC to affect PI3K/AKT signaling and chemoresistance. Our results in this study may provide new insights into a molecular mechanism of regulation of cisplatin-induced apoptosis in human lung cancer cells by nicotine, BDNF, and a β-AR blocker.

## 2. Results

### 2.1. Increased A549 and H1299 Cell Resistance to Cisplatin Was Observed with Nicotine or BDNF Treatment While Opposite Effects Were Found upon Treatment with Propranolol

Studies investigating the effects of tobacco components have used nicotine at concentrations ranging from 10 nM to 10 µM, chosen to be comparable to those found in the bloodstream of smokers, since concentrations of ~200 nM are the average steady state levels in serum, a value that can reach ~ 10 µM or higher immediately after smoking [6,12]. Increased resistance to cisplatin-induced apoptosis was found upon exposure of multiple NSCLC cells to 1 µM nicotine, suggesting that nicotine exposure and activation of nAChR signaling increases lung cancer cell resistance to chemotherapeutic agents [10,14]. 

**Figure 1:**
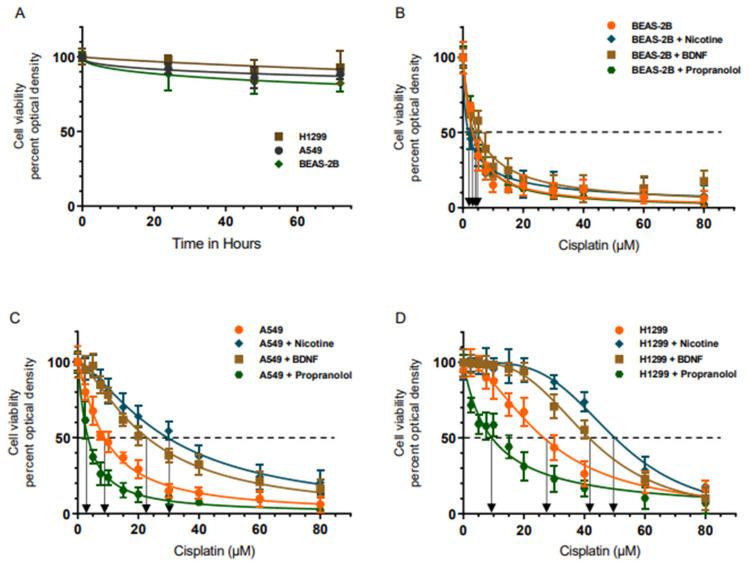
Treatment of A549 and H1299 cells, but not BEAS-2B, with either nicotine or BDNF, increased cell resistance to cisplatin, while the opposite was observed upon treatment with propranolol. Cells were grown to confluence then serum starved overnight. The cell monolayers were then incubated in serum-free media for 72 h in the absence of any treatment (**A**), presence of increasing concentrations of cisplatin without or with nicotine (1 µM), BDNF (5 nM), or propranolol (1 µM) (**B**–**D**). Cell viability of (**A**), BEAS-2B (**B**), A549 (**C**), and H1299 (**D**) was then measured and normalized to cell number (absorbance/cell number), as described in the Methods section. Optical densities (570 nm) were normalized for the curves by expressing each point relative to the best fitted Emax value (set to 100%). The data were then plotted as a function of time or increasing cisplatin concentrations and fit using the GraphPad Prism 9.4.1 software with a nonlinear regression curve fitting approach. Data were expressed as the mean ± S.D. of three independent experiments, each carried out in triplicate.

To investigate the effect of nicotine, BDNF, and propranolol on cell sensitivity to cisplatin, human bronchial epithelial non-cancerous cell line (BEAS-2B), NSCLC A549 and H1299 cells were grown to confluence then serum starved overnight (Figure 1). The cell monolayers were then incubated in serum-free media for 72 h in the absence or presence of increasing concentrations of cisplatin without or with nicotine, BDNF, or propranolol. Cell viability was then measured, as described in the Methods section. Cell viability of the three cell lines was not significantly affected over a period of 72 h (Figure 1A). Cell viability was decreased by treatment with cisplatin in a dose-dependent manner with IC_50_ values of 3.5 ± 0.6 μM for BEAS-2B, 9 ± 1.6 μM for A549, and 27 ± 4 μM for H1299 cells (Figure 1). The IC_50_ values for all three cell lines were consistent with previous reports [4,45,46,47]. No significant effects were observed with any of the treatments of BEAS-2B cells as a function of increasing cisplatin concentrations (Figure 1B). However, cell treatment with nicotine decreased sensitivity to cisplatin of both A549 (Figure 1C) and H1299 (Figure 1D) cell lines (IC_50_ values were 31 ± 5 μM for A549 and 49 ± 8 μM for H1299). A549 and H1299 cell sensitivity to cisplatin also decreased with BDNF treatment (IC_50_ values were 23 ± 3 μM for A549 and 42 ± 7 μM for H1299). Opposite effects were observed when cells were incubated with propranolol (IC_50_ values were 3 ± 0.5 μM for A549 and 9 ± 2 μM for H1299), increasing sensitivity of A549 and H1299 cells to cisplatin.

Since the objective of this work was to examine regulation of cisplatin resistance in lung cancer cells by nicotine, BDNF, and a β-adrenergic receptor blocker, and since no effects were found upon incubation of the non-cancerous cell line, BEAS-2B, with either nicotine, BDNF, or propranolol under our experimental conditions, A549 and H1299 cells were used for the following experiments in this study. 

### 2.2. Increased Cell Viability upon Treatment with Either BDNF or Nicotine Was Reversed by Co-Incubation of Cells with Propranolol in the Absence or Presence of Cisplatin

Noradrenaline, acting via β-AR signaling, is known to have strong stimulating effects on a variety of cancer types and cell treatments with the non-selective β-AR agonist, isoproterenol, leading to increased DNA synthesis of human NSCLC cell lines [7,8,32]. Using NSCLC cell lines, including the H1299 cells employed in this study, cooperation between nAChRs and β-ARs was found to be important in regulating growth stimulation of NSCLC by nicotine [8]. An increase in the neurotransmitter, noradrenaline, accompanied nicotine treatment of NSCLC cells and resulted in increased cell proliferation, effects that were reversed by the α7nAChR antagonist α-BTX or the β-blocker propranolol [8]. 

**Figure 2:**
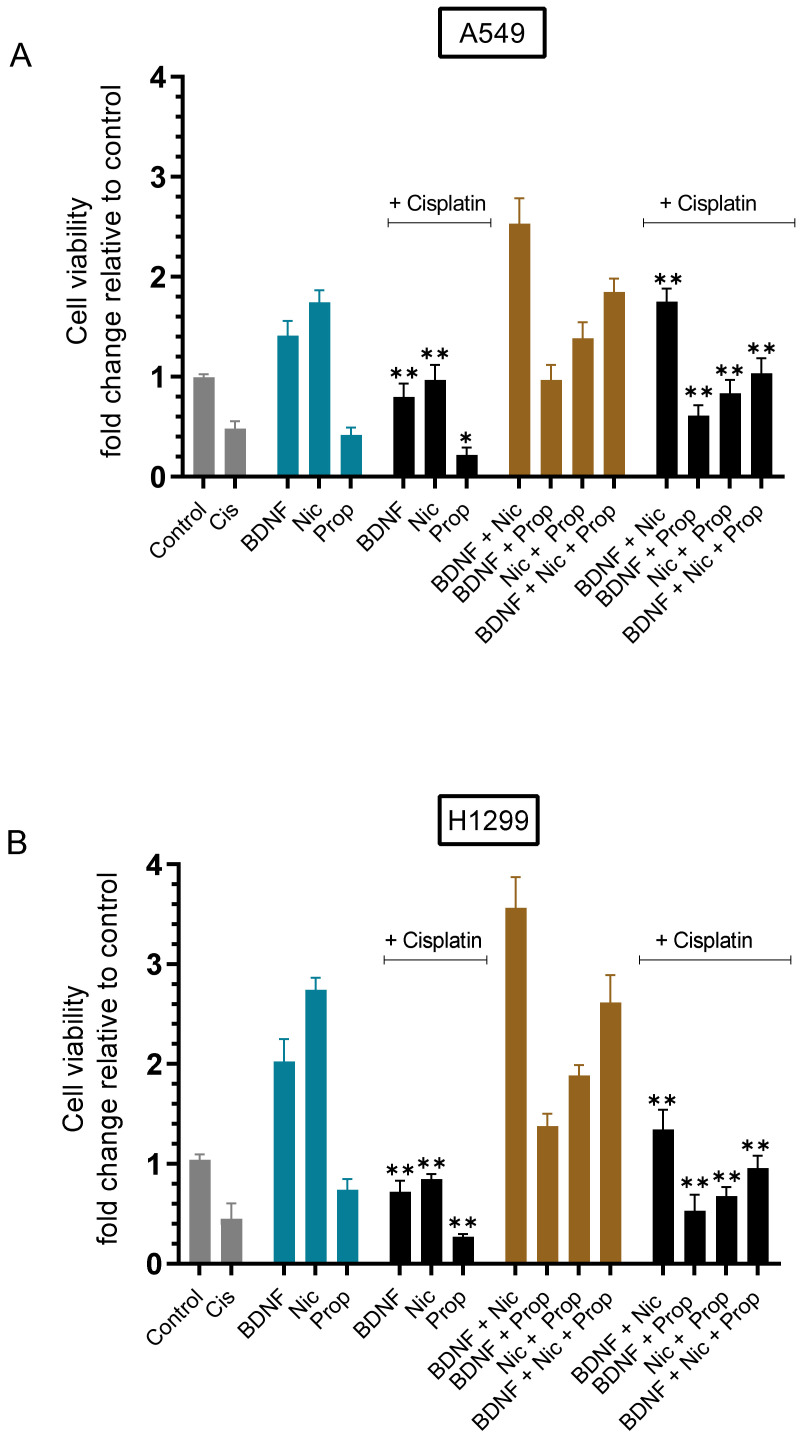
Treatment with propranolol reversed the increase in cell viability measured by treatment with either BDNF or nicotine in the absence or presence of cisplatin. Cells (0.2 × 10^5^) were grown in 10% FBS-supplemented media for 24 h then serum starved overnight. The cell monolayers were then incubated in serum-free media for 72 h in the presence of BDNF (5 nM), nicotine (Nic, 1 µM), and propranolol (Prop, 1 µM), or in combination without or with 10 µM cisplatin when using A549 cells (**A**) and 30 µM cisplatin when using H1299 cells (**B**). Cell viability was then measured as described in the Methods section. Data from five independent assays, each carried out in triplicate, were averaged, normalized, and expressed as fold change relative to untreated cells (control) using the GraphPad 9.4.1 software. The graphs summarize the results expressed as means ± SD (n = 5). Asterisks (*) indicate a statistically significant difference of the cisplatin treated samples compared to the same treatments without cisplatin. Mann–Whitney test, * *p* < 0.05, ** *p* < 0.01.

To examine the effect on viability of cells treated with a combination of BDNF, nicotine, or propranolol, cells were grown in 10% FBS-supplemented media for 24 h then serum starved overnight. The cell monolayers were then incubated in serum-free media for 72 h in the presence of BDNF, nicotine, and/or propranolol without or with cisplatin (Figure 2). Cell viability was then measured, as described in the Methods section. 

Cell viability decreased ~2.00-fold in A549 and H1299 cells treated with cisplatin (Figure 2A,B). Treatment of A549 cells with BDNF or nicotine led to ~1.40-fold and ~1.75-fold increases in viability, respectively (Figure 2A). BDNF treatment of H1299 cells led to ~2.00-fold increase in cell viability, while an increase of ~2.75-fold in viability was measured upon cell treatment with nicotine (Figure 2B). Cell treatment with propranolol resulted in ~1.35-fold decrease in H1299 cell viability and a ~2.40-fold decrease in the viability of A549 cells (Figure 2). Both BDNF and nicotine were partially able to rescue against cisplatin-induced cytotoxicity in both cell lines, while the opposite effects were found by cell treatment with propranolol, which augmented the effects of cisplatin on cell viability in both cell lines (Figure 2). Relative to the untreated control, co-treatment of A549 cells with BDNF and nicotine increased cell viability by ~2.55-fold, a value higher than that measured for treatment with either BDNF or nicotine alone (Figure 2A). Similarly, viability of H1299 cells increased by ~3.55-fold relative to untreated control upon a combined treatment with both BDNF and nicotine compared to the single treatments (Figure 2B). A549 and H1299 cell treatments with propranolol and BDNF, nicotine, or BDNF and nicotine, decreased cell viability relative to treatments in the absence of propranolol, effects that were further enhanced upon addition of cisplatin with similar trends (Figure 2). 

### 2.3. Cell Treatment with Epinephrine or Nicotine Led to Increased EGFR Activation Effects, Opposite to Those Found with Propranolol, Blocking EGFR Activation by Erlotinib Increased Cell Sensitivity to Cisplatin

Nicotine’s effect likely involves the epidermal growth factor receptors (EGFRs), since it has been previously reported that nicotine induces secretion of growth factors leading to tumor progression and metastasis [7]. Binding of nicotine to α7nAChRs can induce the secretion of growth factors, such as EGF, IGF, along with neurotransmitters, such as adrenaline and noradrenaline [7,48]. This nicotine-mediated increased secretion of EGF and IGF leads to the transactivation of EGFR and IGF-1R, resulting in the activation of mitogenic and antiapoptotic pathways [7,48]. Moreover, release of adrenaline and noradrenaline, the physiological ligands for β-AR, upon binding of nicotine to α7nAChRs, activates the β-AR which, in turn, stimulates release of EGF and IGF, thereby promoting growth and proliferation contributing to lung cancer progression [7,48]. Constitutive activation of EGFR has been reported to be associated with poorer prognosis in patients with NSCLC [49,50,51]. Erlotinib is a synthetic anilinoquinazoline and orally selective EGFR tyrosine kinase inhibitor that leads to decreased survival of cancer cells [51].

**Figure 3:**
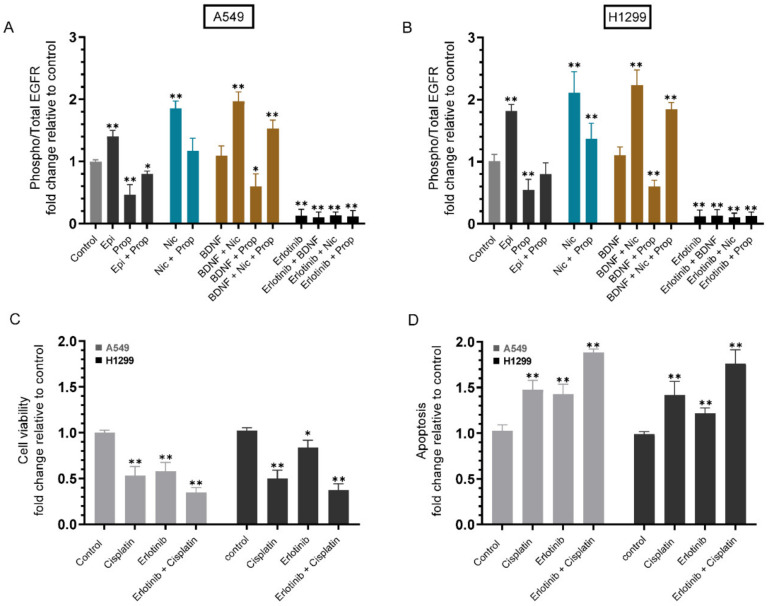
Phospho/Total EGFR ratio increased by cell treatment with epinephrine or nicotine and decreased by propranolol, while blocking EGFR activation by erlotinib increased cell sensitivity to cisplatin. Cells (0.2 × 10^5^) were grown in 10% FBS-supplemented media for 24 h. The following day, the cell monolayers were serum starved for 24 h, then incubated in serum-free media for 72 h in the presence of epinephrine (Epi, 100 nM), propranolol (Prop, 1 µM), nicotine (Nic, 1 µM), BDNF (5 nM), erlotinib (10 µM), or in combination. The phospho/total EGFR assay (**A**,**B**) was carried out as described in the Methods section. Cell viability (**C**) and apoptosis (**D**) were determined in the presence of erlotinib (10 µM) without or with 10 µM cisplatin, when using A549 cells, and 30 µM cisplatin, when using H1299 cells, as described in the Methods section. Data from five independent assays, each carried out in triplicate, were averaged, normalized, and expressed as fold change relative to control untreated cells (Control) using the GraphPad 9.4.1 software. The graphs summarize the results expressed as means ± SD (n = 5). Asterisks (*) indicate a statistically significant difference from the corresponding control for each cell line, Mann–Whitney test, while the absence of asterisks indicates no significance. * *p* < 0.05, ** *p* < 0.01.

To examine the effects of nicotine, epinephrine, propranolol, and BDNF, on the phospho/total EGFR ratio, and the outcome of blocking EGFR activation on cell sensitivity to cisplatin, cells were grown in 10% FBS-supplemented media for 24 h. The following day, the cell monolayers were serum starved for 24 h, then incubated in serum-free media for 72 h in the presence of epinephrine, propranolol, nicotine, BDNF, erlotinib, or in combination (Figure 3). 

Treatment with epinephrine increased the phospho/total EGFR ratio ~1.40-fold in A549 cells (Figure 3A) and ~1.80-fold in H1299 cells (Figure 3B). This ratio was decreased ~2.00-fold in A549 cells treated with propranolol, an effect that was partially reversed by co-treatment of A549 cells with epinephrine and propranolol (~1.25-fold decrease) (Figure 3A). Similar trends were observed for H1299 cells with a decrease in the phospho/total EGFR ratio of ~1.85-fold with propranolol treatment and ~1.25-fold decrease upon co-treatment with epinephrine and propranolol (Figure 3B). In both cell lines, the ratio of phospho/total EGFR was activated by nicotine treatment (A549, ~1.85-fold and H1299, ~2.00-fold). This activation was reduced to ~1.15-fold and to ~1.35-fold in A549 and H1299, respectively, when cells were co-treated with nicotine and propranolol (Figure 3A,B). Compared to the effects observed by using epinephrine or nicotine, smaller effects were found on the ratio of phospho/total EGFR upon cell incubation with BDNF in either cell line. However, treatment of cells with BDNF, nicotine, and propranolol led to an increase (~1.50-fold in A549 and ~1.85-fold in H1299) in the phospho/total EGFR ratio, values that were higher than those found upon treatment with nicotine and propranolol (~1.15-fold increase in A549 and ~1.35-fold increase in H1299) (Figure 3A,B). These results might suggest a role of BDNF in affecting the phospho/total EGFR ratio under these conditions. 

Treatment of cells with the EGFR inhibitor, erlotinib, decreased A549 cell viability ~1.70-fold and increased apoptosis ~1.45-fold, while a ~1.20-fold change was found on cell viability or apoptosis in H1299 cells (Figure 3C,D). Co-treatment with cisplatin and erlotinib led to a decrease in A549 cell viability of ~2.85-fold and an increase in apoptosis of ~1.85-fold. A similar trend was observed with cisplatin and erlotinib co-treatment when using H1299 cells with a decrease of ~2.60-fold in cell viability and an increase in apoptosis of ~1.75-fold (Figure 3C,D). 

### 2.4. Phospho/Total IGF-1R Ratio Increased upon Cell Treatment with Epinephrine or Nicotine and Decreased by Propranolol, While Cell Sensitivity to Cisplatin Increased by Blocking IGF-1R Activation

Insulin-like growth factor 1 receptor (IGF-1R) is a transmembrane heterotetramer receptor protein tyrosine kinase which consists of two α-subunits and two β-subunits linked by disulfide bonds [52,53]. Ligand binding to the receptor results in receptor oligomerization, activation, and phosphorylation of cellular substrates that consequently leads to cellular proliferation [53,54]. IGF-1R is implicated, and often overexpressed, in a variety of human cancers, including lung cancer [55]. IGF-1R is recognized to play a key role in tumorigenesis, and protection of tumor cells from apoptosis triggered by chemotherapy [53]. Increased activation of IGF-1R and its downstream signaling resulted from treatment of normal bronchial epithelial cells with norepinephrine and led to cellular transformation, an effect that was blocked by cell treatment with the β-blocker, propranolol [31].

**Figure 4:**
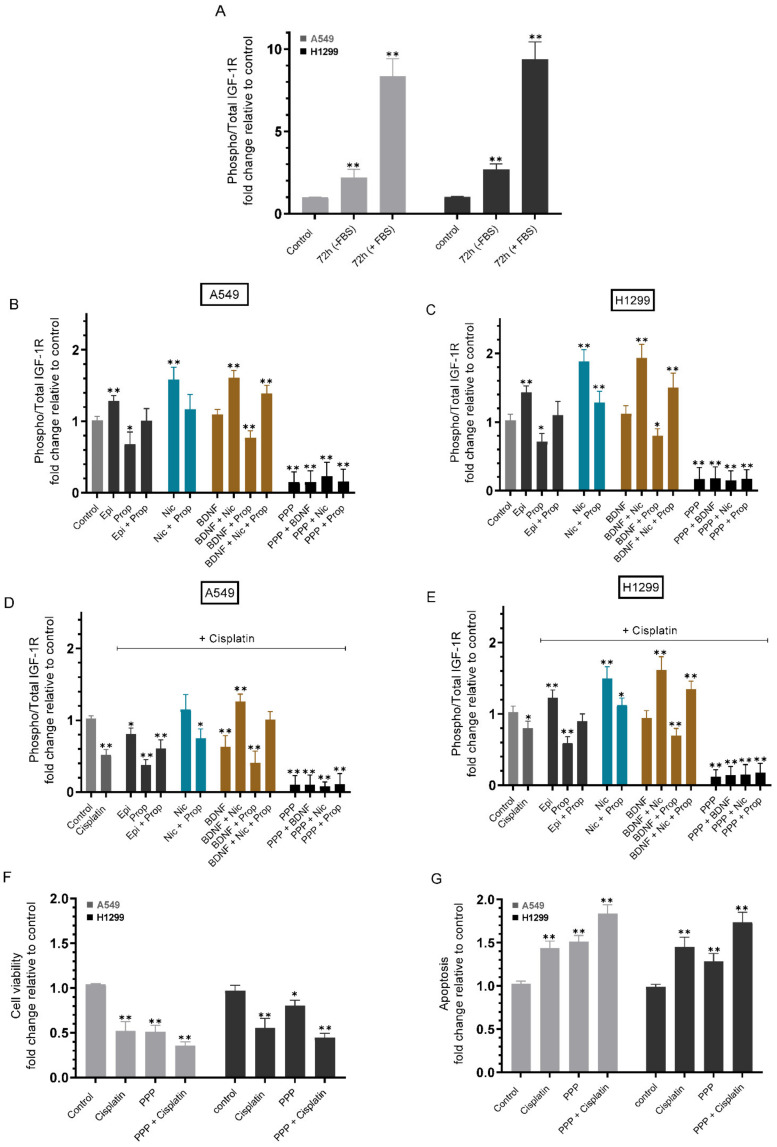
Phospho/Total IGF-1R ratio increased by cell treatment with epinephrine or nicotine and decreased by cell treatment with propranolol, while blocking IGF-1R activation by PPP increased cell sensitivity to cisplatin. (**A**) Cells (0.2 × 10^5^) were grown in 10% FBS-supplemented media for 24 h. The following day, the cell monolayers were incubated in serum-free media for 24 h (Control), then incubated without or with FBS for 72 h. The phospho/total IGF-1R assay (**A**) was carried out on the same amount of protein (25 µL of 400 µg/mL total protein) of the cell lysate as described in the Methods section. The phospho/total IGF-1R assay was also carried out on A549 and H1299 cells grown as above then incubated in serum-free media for 72 h in the absence or presence of epinephrine (Epi, 100 nM), propranolol (Prop, 1 µM), nicotine (Nic, 1 µM), BDNF (5 nM), PPP (5 µM) or in combination without (**B**,**C**) or with cisplatin (**D**,**E**). Cell viability (**F**) and apoptosis (**G**) were determined in the presence of PPP (5 µM) without or with 10 µM cisplatin when using A549 cells and 30 µM cisplatin when using H1299 cells, as described in the Methods section. Data from five independent assays, each carried out in triplicate, were averaged, normalized, and expressed as fold change relative to untreated control cells (Control) using the GraphPad 9.4.1 software. The graphs summarize the results expressed as means ± SD (n = 5). Asterisks (*) indicate a statistically significant difference from the corresponding control for each cell line, Mann–Whitney test, while the absence of asterisks indicates no significance. * *p* < 0.05, ** *p* < 0.01.

We tested the activation of the IGF-1R in the absence or presence of FBS (Figure 4A). The phospho/total IGF-1R assay was carried out on the same amount of protein of the cell lysate as described in the Methods section. Compared to control cells, the ratio of phospho/total IGF-1R in A549 cells was ~2.20-fold higher in the absence of FBS and 8.35-fold higher in the presence of FBS, while the ratio in H1299 cells increased ~2.65-fold in the absence of FBS and ~9.35-fold in the presence of FBS (Figure 4A). 

To test the effect of cell treatment with epinephrine, nicotine, BDNF, or propranolol, without or with cisplatin, on activation of the IGF-1R, cells were grown in 10% FBS-supplemented media for 24 h followed by serum starvation overnight. The cells were then incubated in serum-free media for 72 h in the absence or presence of epinephrine, propranolol, nicotine, BDNF, and/or picropodophyllin (PPP), a potent, competitive cyclolignan compound, and reversible selective inhibitor of the IGF-1R that leads to downregulation of the PI3K/Akt signaling cascade [56]. Treatment of A549 cells with epinephrine increased the ratio of phospho/total IGF-1R by ~1.25-fold while an increase of ~1.45-fold was found in H1299 cells (Figure 4B,C). Treatment with epinephrine and propranolol diminished the inhibitory effects on IGF-1R activation in cells treated with only propranolol that showed a decrease of ~1.45-fold and ~1.40-fold in the activity of IGF-1R in A549 and H1299 cells, respectively, compared to control untreated cells. Cell incubation with nicotine increased the ratio of phospho/total IGF-1R ~1.55-fold in A549 cells and ~1.85-fold in H1299 cells. This increase was partially negated by co-incubation of cells with nicotine and propranolol, decreasing the ratio to ~1.15-fold in A549 cells and ~1.25-fold in H1299 cells (Figure 4B,C). While treatment of either cell line with BDNF did not show a significant change on the activation of IGF-1R compared to control, co-treatment of A549 cells with a combination of BDNF, nicotine, and propranolol resulted in ~1.40-fold increase in the phospho/total IGF-1R ratio, compared to ~1.15-fold increase upon cell treatment with a combination of nicotine and propranolol. Similarly, the ratio of phospho/total IGF-1R increased ~1.5-fold when H1299 cells were co-treated with a combination of BDNF, nicotine, and propranolol and ~1.25-fold when cells were co-treated with nicotine and propranolol. These results might indicate a function of BDNF in regulating the activation of IGF-1R in these cell lines. The values for the ratio of phospho/total IGF-1R were lower with the same treatments in the presence of cisplatin (Figure 4D,E), but had a comparable trend as that observed in the absence of cisplatin (Figure 4B,C).

Cell viability and apoptosis were determined in the presence of PPP without or with cisplatin (Methods). Blocking activation of IGF-1R with PPP decreased A549 cell viability by ~2.00-fold and increased apoptosis by ~1.50-fold, while ~1.25-fold change was observed for H1299 cells treated with PPP (Figure 4F,G). A reduction in A549 cell viability (~2.85-fold decrease) was found upon co-incubation with both PPP and cisplatin and correlated with ~1.85-fold increase in apoptosis. Co-incubation of H1299 cells with PPP and cisplatin led to ~2.20-fold decrease in viability and ~1.70-fold increase in apoptosis (Figure 4F,G), effects that were greater than those obtained when cells were treated with only cisplatin or PPP. 

### 2.5. The PI3K and AKT Activities Were Upregulated by Nicotine or BDNF and Downregulated by Cell Treatment with Erlotinib, PPP, or Propranolol

The effects of nicotine primarily occur through its binding to, and activation of, cell surface nAChRs, in particular, the α7-nAChRs, stimulating a variety of cancer-inducing signaling cascades, including the PI3K/AKT pathway, increasing cell proliferation, promoting tumor progression, and resistance to apoptosis induced by different agents, significantly contributing to the oncogenic process [6,7,11,18,25]. BDNF is known to mediate NSCLC tumor growth via activation of the PI3K/AKT signaling pathway [57,58,59]. Using inhibitors, we recently reported that the levels of BDNF in the media of A549 and H1299 cells are likely regulated by PI3K/AKT signaling [40]. The PI3K/AKT pathway is known to be upregulated by activation of EGFR and IGFR in NSCLC [16,17,60]. 

**Figure 5:**
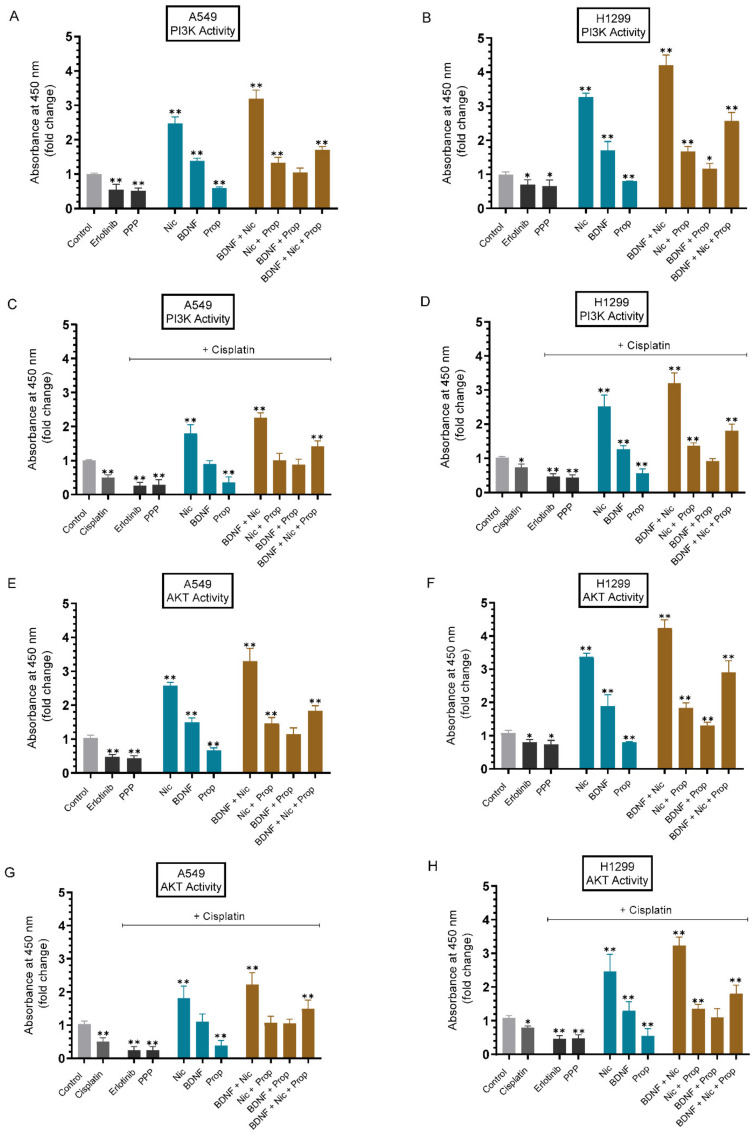
The activities of PI3K and AKT were upregulated by cell treatment with nicotine or BDNF and blocked by erlotinib, PPP, and propranolol. Cells (0.2 × 10^5^) were grown in 10% FBS-supplemented media for 24 h then serum starved overnight. The cells were then incubated in serum-free media without or with cisplatin for 72 h in the presence of erlotinib (10 µM), PPP (5 µM), nicotine (Nic, 1 µM), BDNF (5 nM), propranolol (Prop, 1 µM), and in combination. The PI3K activity (**A**–**D**) was assayed by the Total In-Cell ELISA Kit and the AKT activity (**E**–**H**) was measured on the same amount of protein (3 µL of 600 µg/mL total protein) of the cell lysate, as described in the Methods section. Data from five independent assays, each carried out in triplicate, were averaged, normalized, and expressed as fold change relative to untreated cells (control) using the GraphPad 9.4.1 software. The graphs summarize the results, expressed as means ± SD (n = 5). Asterisks (*) indicate a statistically significant difference from the control for each cell line, Mann–Whitney test, while the absence of asterisks indicates no significance. * *p* < 0.05, ** *p* < 0.01.

Cells were grown in 10% FBS-supplemented media for 24 h then serum starved overnight. The cells were then incubated in serum-free media for 72 h without or with cisplatin in the presence of erlotinib, PPP, nicotine, BDNF, propranolol, and in combination, to examine the effect of these treatments on the PI3K and AKT activities, measured as described in the Methods section. Treatment of cells with erlotinib or PPP decreased the PI3K activity ~1.80-fold (Figure 5A) in A549 cells and ~1.50-fold in H1299 cells (Figure 5B). The values for the PI3K activity were lower with the same treatments in the presence of cisplatin (Figure 5C,D). The same treatments resulted in decreased AKT activity (~2.15-fold decrease in A549 cells (Figure 5E) and ~1.30-fold decrease in H1299 (Figure 5F) cells). Similar to the results obtained for PI3K, the AKT activities were lower with the same treatments in the presence of cisplatin (Figure 5G,H). Increased activation of PI3K was found upon cell treatment with nicotine (A549 ~2.45-fold, H1299 ~3.30-fold), BDNF (A549 ~1.40-fold, H1299 ~1.70-fold) (Figure 5A,B). Comparable levels of AKT activation were found under the same conditions (Figure 5E,F). Treatment of A549 cells with propranolol decreased PI3K activation ~1.65-fold (Figure 5A) and AKT activation (Figure 5E) ~1.50-fold. Decreased PI3K and AKT activation of ~1.25-fold (Figure 5B,F) was found in H1299 cells treated with propranolol. A549 and H1299 cell incubation with either nicotine or BDNF and propranolol decreased the effects of nicotine or BDNF on PI3K and AKT activation (Figure 5). Co-treatment of either A549 or H1299 cells with BDNF and nicotine led to a greater increase in the PI3K and AKT activities, compared to treatment with either BDNF or nicotine alone. This increased kinase activation was blocked by cell incubation with BDNF, nicotine, and propranolol (Figure 5). In the presence of cisplatin, the values for the PI3K (Figure 5C,D) and AKT (Figure 5G,H) activities were lower with the same treatments, but had a comparable trend as that observed in the absence of cisplatin.

### 2.6. Co-Treatment of Cells with Inhibitors against PI3K and AKT Increased Cell Sensitivity to Cisplatin

Nicotine is the major component in tobacco and as a survival agonist, was previously shown to negatively impact apoptosis induced by the chemotherapeutic agent cisplatin, with a key role of α5-nAChR/AKT signaling in this process in human gastric cancer cell lines [13]. The PI3K/AKT pathway is activated by nicotine and is considered a major cascade associated with cancer [7,61]. The pathway is known to regulate cell survival and has been linked to induction of chemoresistance [7,61]. Evasion of apoptosis is a major mechanism responsible for cisplatin resistance [2]. In lung cancer cells, nicotine was shown to inhibit apoptosis induced by cisplatin through a mechanism mediated by nAChRs and which, in part, involves activation of PI3K/AKT and PKC/ERK signaling [10,14]. Co-treatment with siRNA-α5-nAChR and LY294002, a PI3k inhibitor, increased apoptosis induced by cisplatin and blocked the antagonizing effects of nicotine on cisplatin cytotoxicity in a human gastric cancer cell line [13]. Treatment with BDNF attenuated cisplatin-induced cell death and decreased the chemosensitivity of neuroblastoma cells to cisplatin, an effect that was blocked by cell treatment with the PI3K inhibitor, LY294002, suggesting a role of PI3K in BDNF’s rescue effects [41].

**Figure 6:**
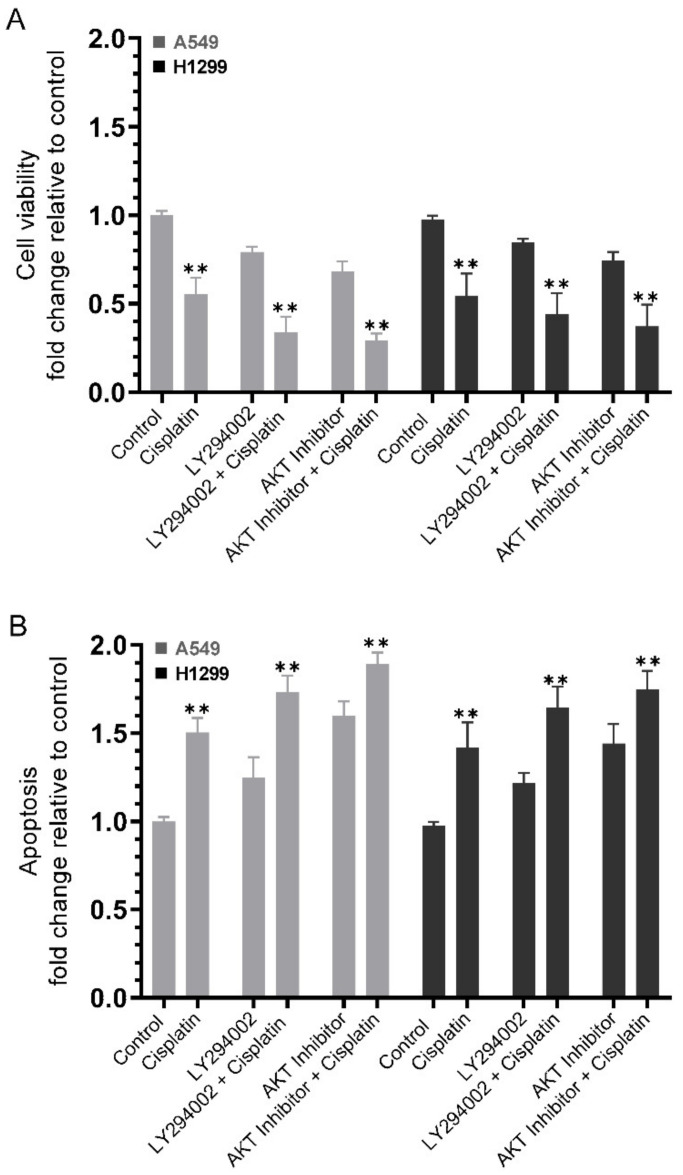
Cell sensitivity to cisplatin increased upon co-treatment of cells with inhibitors against PI3K and AKT. Cells (0.2 × 10^5^) were grown in 10% FBS-supplemented media for 24 h. The following day, the cell monolayers were incubated in serum-free media for 24 h, then treated as indicated for 72 h with inhibitors targeted against PI3K (LY294002, 14.5 μM) and AKT (AKT inhibitor, 1.75 μM) without or with 10 μM cisplatin when using A549 cells and 30 μM cisplatin when using H1299 cells. Cell viability (**A**) and apoptosis (**B**) were then determined as described in the Methods section. Data from five independent assays, each carried out in triplicate, were averaged, normalized, and expressed as fold change relative to untreated cells (Control) using the GraphPad 9.4.1 software. The graphs summarize the results expressed as means ± SD (n = 5). Asterisks indicate a statistically significant difference from the corresponding samples without cisplatin treatment for each cell line, Mann–Whitney test, ** *p* < 0.01.

Our results (Figure 5) showed upregulation of the PI3K and AKT activities by nicotine or BDNF and downregulation by cell treatment with erlotinib, PPP, or propranolol. To test the effects of inhibiting the activities of PI3K and AKT on cell viability or apoptosis in the absence or presence of cisplatin, cells were grown in 10% FBS-supplemented media for 24 h then serum starved overnight. The cells were then treated as indicated for 72 h with inhibitors targeted against PI3K and AKT without or with cisplatin. Treatment of A549 cells with LY294002 and cisplatin reduced cell viability ~3.00-fold, a decrease greater than that found upon cell treatment with either LY294002 (~1.25-fold decrease) or cisplatin (~1.80-fold decrease) (Figure 6A). These results correlated with a ~1.75-fold increase in apoptosis upon co-treatment of A549 cells with LY294002 and cisplatin, versus an increase of ~1.50-fold and 1.25-fold with cisplatin or LY294002 treatment, respectively (Figure 6B). Similar trends were observed using H1299 cells with a decrease in cell viability of ~1.85-fold with cisplatin treatment, ~1.20-fold decrease with LY294002 treatment, and ~2.27-fold decrease with cisplatin and LY294002 co-treatment (Figure 6A). The differences in apoptosis correlated with those of H1299 cell viability under these conditions with an increase of ~1.40-fold in apoptosis with cisplatin treatment, ~1.20-fold increase with LY294002 treatment, and ~1.65-fold with the combined treatment (Figure 6B). Comparable effects to those observed upon blocking PI3K activity were found using the AKT inhibitor. Cell treatment with the AKT inhibitor decreased A549 cell viability (~1.45-fold and ~3.45-fold without or with cisplatin, respectively) and H1299 cell viability (~1.35-fold and ~2.70-fold without or with cisplatin, respectively) (Figure 6A). These values correlated with increased apoptosis upon A549 cell treatment with the AKT inhibitor (increase of ~1.60-fold and ~1.90-fold without or with cisplatin, respectively). Similarly, H1299 cell treatment with the AKT inhibitor led to ~1.45-fold increase in apoptosis, while co-treatment with the AKT inhibitor and cisplatin resulted in ~1.75-fold increase in apoptosis (Figure 6B).

## 3. Discussion

For both men and women, the leading cause of cancer-related deaths worldwide is lung cancer, which far exceeds other cancers, such as those of the prostate, breast, and colon [1]. NSCLC, which represents ~80% of all lung cancer cases, is highly resistant to current cancer therapeutics [9]. 

The strongest documented risk factor for lung cancer development is cigarette smoke, with a significant proportion of NSCLC cases detected after the onset of metastasis [62,63]. Of the complex mixture of more than 4000 compounds in cigarette smoke, nicotine has been reported to be the major addictive component [63]. Tobacco smoke has been classified by the IARC as a Group 1 carcinogen [64]. While nicotine has been shown not to be carcinogenic, it is able to induce proliferation, growth stimulation of NSCLC, and progression of tumors, contributing to the ability of cancer cells to evade apoptosis in a range of experimental models at concentrations of 10^−8^ M to 10^−7^ M, normally found in the bloodstream of smokers [6,7,11,25]. Exposure of multiple NSCLC cells to 1 µM nicotine was previously shown to increase resistance to cisplatin-induced apoptosis, indicating that lung cancer cell resistance to chemotherapeutic agents could be enhanced by nicotine exposure and activation of nAChR signaling [10,14]. In investigating the effect of nicotine, BDNF, and propranolol, on A549 and H1299 cell sensitivity to cisplatin, we found increased cell resistance to cisplatin with nicotine or BDNF treatment, while opposite effects were found upon treatment with propranolol (Figure 1). The IC_50_ values were generally greater when using H1299 cells, as compared to A549 cells (Figure 1). This finding might be explained, in part, by previous studies using the lung cancer cell lines H1299 (p53^−/−^) and A549 (p53^+/+^), showing that nicotine induces more proliferation in lung cancer cells lacking p53 and that nicotine-dependent induction of survival signaling in lung cancer cells was dependent on p53, while curcumin completely blocked the over-expression of these survival pathways via p53-independent mechanisms [62]. In addition, treatment of H1299 cells, but not A549 cells, with lower concentrations of nicotine, were sufficient to induce NFκB activation, while a ~100X higher concentration of nicotine was needed to induce activation of NFκB in A549 cells [62]. 

A number of non-neuronal cells have been recently shown to synthesize and release ACh and, in addition, norepinephrine and epinephrine in response to autostimulation by ACh or upon exposure to exogenous agonists of nAChRs [7,8,31,32,48]. Nicotine was shown earlier to mediate increased mitogenic signaling through activation of β-AR in addition to the nAChRs [7,8]. Blocking β-ARs was found to increase cisplatin sensitivity of human lung cancer cells in vitro [32]. Use of β-blockers is thought to be a promising pharmacological intervention that may lead to lowered risk of NSCLC development [7,8]. Our results showed increased cell viability upon treatment with either BDNF or nicotine, but that co-incubation of cells with propranolol reversed this effect on cell viability in the absence or presence of cisplatin (Figure 2). 

In addition to the β-ARs, EGFR and IGFR are also thought to play an important role in chemoresistance [7,48]. Nicotine was shown to stimulate secretion of epinephrine and norepinephrine, which then bind β-AR, leading to activation of downstream oncogenic signaling cascades, including the EGFR, IGFR, and the PI3K/AKT pathways [7,8,32,48]. Several tyrosine kinase inhibitors that target EGFR, such as geftinib, have been shown to enhance the inhibitory effect of cisplatin through disruption of the EGFR signaling cascade, and improve survival of NSCLC patients receiving platinum-based chemotherapy [2,3]. A functional network and cooperative interplay have been suggested involving nAChRs, β-AR, EGFR, and IGFR, all known to be expressed, and frequently found to co-exist in human lung cancer cells, likely leading to increased mitogenic effects enhancing oncogenesis [7,48]. The tobacco-specific carcinogen 4-(methylnitrosamino)-1-(3-pyridyl)-1-butanone (NNK) was shown to activate the IGF-1R, via the β-Ars, leading to increased lung carcinogenesis [55]. Agonists of β-ARs were found to increase phosphorylation of IGF-1R, while β-AR antagonists suppressed NNK-induced IGF-1R phosphorylation and lung tumor formation in mice [55]. Chemosensitivity to cisplatin was shown to be enhanced in A549 cells upon knockdown of IGF-1R [52]. 

Cell treatment with epinephrine or nicotine led to increased EGFR activation, effects opposite to those found with propranolol (Figure 3). Moreover, blocking EGFR activation using erlotinib increased cell sensitivity to cisplatin in both cell lines (Figure 3). Similarly, the phospho/total IGF-1R ratio increased upon cell treatment with epinephrine or nicotine and decreased by propranolol, while cell sensitivity to cisplatin increased by blocking IGF-1R activation (Figure 4). 

Activation of the membrane ligand-gated ion channels, nAChRs, by nicotine contributes to anti-apoptosis processes, cancer progression, and drug resistance in lung cancer, in part, due to activation of the PI3K/AKT signaling cascade [65]. Ligand binding to EGFR is also known to induce tumor progression through activation of downstream pathways, including PI3K/AKT [60,66]. In addition, upregulation of PI3K/AKT signaling is known to result from activation of EGFR and IGFR in NSCLC [16,17,60]. Moreover, BDNF is known to mediate its effects on NSCLC tumor growth via activation of the PI3K/AKT signaling pathway [57,58,59]. Using inhibitors, we previously reported that the levels of BDNF in the media of A549 and H1299 cells are regulated by the PI3K/AKT signaling pathway [40]. Our results from this study showed that the PI3K and AKT activities were upregulated by nicotine or BDNF, and downregulated by cell treatment with erlotinib, PPP, and propranolol (Figure 5). 

The PI3K/AKT pathway is activated by nicotine and has been linked to induction of chemoresistance, in part due to evasion of apoptosis [7,61]. BDNF treatment abolished cisplatin-induced cell death and reduced the chemosensitivity of neuroblastoma cells to cisplatin, an effect that was blocked by treatment of cells with the PI3K inhibitor, LY294002, indicating a role of PI3K in BDNF’s rescue effects [41]. Much controversy exists over the stress–cancer connection and a mechanism linking stress hormones to tumors was shown to be due to direct effects of epinephrine in protecting cancer cells from apoptosis [67]. Agonists of GPCRs were reported to decrease apoptosis in prostate cancer cells, in part, via the PI3K//AKT signaling pathway [7,31]. Our data showed that co-treatment of A549 and H1299 cells with inhibitors against PI3K or AKT increased cell sensitivity to cisplatin (Figure 6). 

**Figure 7:**
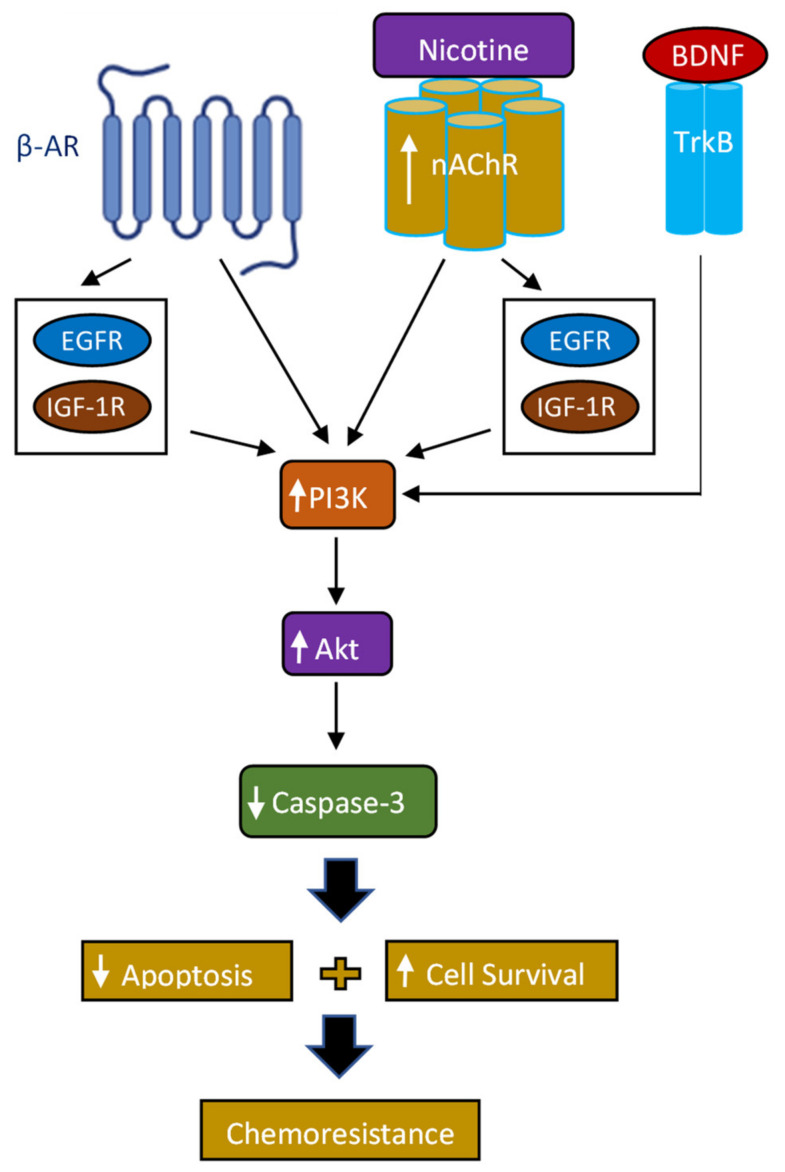
Representation of the main hypothesis and findings of this study.

Nicotine is known to be able to bind to β-ARs and can also trigger the production of β-AR ligands, such as adrenaline and noradrenaline, leading to activating β-ARs, promoting cancer progression and inhibiting apoptosis induced by cisplatin in lung cancer cells [7,8,14,65]. Binding of nicotine to nAChRs mediates increased secretion of EGF and IGF which leads to the transactivation of EGFR and IGF-1R signaling, thus, contributing to cell survival signaling cascades and anti-apoptotic pathways [7,48,68]. Invasion of A549 cells was shown to be inhibited upon blocking receptor TrkB signaling [69]. Nicotine has been shown to accelerate cell proliferation by increasing the levels of growth factor receptors and growth factors. including BDNF [11,12]. Nicotine is a known exogenic activator for BDNF release in the extracellular matrix [11,12,70]. The levels of BDNF were reported to increase under external stimulators, such as nicotine, which was reported to induce release of BDNF from neuroblastoma cancer cells (SH-SY5Y) [70]. Based on our results, we proposed a model (Figure 7) summarizing the main hypothesis and findings of this study. How nicotine affects the levels of adrenaline, noradrenaline, EGF, IGF, and BDNF under different cellular conditions is the main current research focus of our laboratory. 

A promising strategy in cancer therapy to combat cancers, such as NSCLC, includes targeting molecules critical to cancer development by a combination therapy using two or more therapeutic agents [2,3]. Our findings shed light on an interplay between nicotine, BDNF, and β-AR signaling in regulating lung cancer cell survival and chemoresistance, which could likely lead to the development of novel therapeutic strategies that target this regulatory network in the future.

## 4. Materials and Methods

### 4.1. Materials

Most of the materials used in this study were purchased as we reported earlier [40,71,72,73,74]. Phosphate Buffered Saline (PBS), LY294002 hydrochloride, hydrogen peroxide solution, nicotine, BDNF, epinephrine, propranolol hydrochloride, erlotinib, picropodophyllin (PPP), and AKT Inhibitor (Calbiochem, San Diego, CA, USA) were purchased from Sigma-Aldrich (St. Louis, MO, USA). The caspase 3 (cleaved) colorimetric In-Cell ELISA Kit (62218), the BCA protein assay kit, and the Halt Protease and Phosphatase Inhibitor Cocktail were from ThermoFisher (Waltham, MA, USA). 

### 4.2. Cell Culture

Human bronchial epithelial cells, BEAS-2B (ATCC CRL-9609), human NSCLC cell lines, A549 (ATCC CCL-185) and H1299 (ATCC CRL-5803), were purchased from the American Type Culture Collection (ATCC, Manassas, VA, USA). BEAS-2B were cultured in LHC-9 media according to the recommendation by ATCC. A549 and H1299 cells were seeded, as we reported earlier [40,71,72,73,74,75,76], in 5 mL HyClone Dulbecco’s modified Eagle’s media/nutrient mixture F-12 (DMEM/F12) (GE Healthcare Life Sciences, Pittsburgh, PA, USA), supplemented with 10% Fetalgro bovine growth serum (FBS, RMBIO, Missoula, MT, USA), 50 U/mL penicillin, and 50 U/mL streptomycin (Invitrogen Life Technologies, Carlsbad, CA, USA) in 25 cm^2^ tissue culture flasks, and allowed to grow overnight in an incubator at 37 °C, 95% humidity, and 5% CO_2_. The cells were counted after trypan blue staining, with a hemocytometer. 

### 4.3. MTT Assay

The MTT reduction assay (Sigma-Aldrich), used to measure cell viability, was carried out as we reported earlier [71,74,77]. Cells were seeded in 96-well plates in 200 μL 10% FBS-supplemented media per well and maintained overnight at 95% humidity and 5% CO_2_. After an overnight incubation, the media was replaced with 200 μL serum-free media and the cells were further incubated, without or with different treatments, for 24, 48, or 72 h. The final concentration of DMSO in each well, never exceeded 0.1%. The cells were then incubated for 4 h with MTT (0.5 mg/mL) in the dark. The media was carefully removed and DMSO (100 μL) was added to dissolve the formazan crystals. The absorbance was measured at 570 nm in a plate reader and normalized to cell number (absorbance/cell number). All absorbance measurements were in the linear range. Untreated cells, or wells containing only DMSO and media, were used as a positive and negative control, respectively. Statistical analysis was conducted using GraphPad Prism version 9.4.1 for Windows. Significant values were considered at *p* < 0.05 and more significant values at *p* < 0.01, compared with the control. 

### 4.4. Apoptosis Assay

For the caspase 3 (cleaved) colorimetric assay, activated (cleaved) caspase 3 and tubulin were simultaneously measured in triplicate in whole cells, by an in-cell ELISA assay (62218, ThermoFisher), using 96-well microplates, as we previously described [77,78]. Briefly, cells were plated per well and incubated overnight at 37 °C in 5% CO_2_. Cells were treated as indicated then subsequently fixed with 4% formaldehyde, permeabilized, according to the manufacturer’s instructions, and incubated with primary antibodies overnight at 4 °C. HRP conjugates were added next and incubated at RT for 30 min. Subsequent to washing the plates, the TMB substrate was added to each well and incubated in the dark at RT. The reaction was typically stopped after 15 min with a TMB stop solution when the blue color became apparent. The absorbance was then measured at 450 nm within 30 min after the reaction was stopped. Control cells were treated with a 0.1% DMSO vehicle control and contained all the reagents except the primary antibodies. The average of all replicate nonspecific background signal controls from each condition was subtracted and then the average absorbance at 450 nm for each condition was calculated.

### 4.5. Cisplatin Synthesis

Cisplatin was synthesized by the Dhara method [79], where 0.415 g of K_2_[PtCl_4_] (Sigma Aldrich) was reacted with 8.0 mL of a saturated aqueous solution of potassium iodide. After stirring, 2.20 mL of cold ammonium hydroxide was added dropwise and allowed to react until the mother liquor became a pale-yellow color. The solid formed was filtered, washed with water, and dried. Next, 2.20 mL of 1 M silver nitrate was added, and the reaction heated for 20 min at 40 °C, sonicating once immediately after the addition and once ten minutes into the heating, forming a green precipitate which was filtered using minimal water, and the solid was discarded. To the filtrate, 0.8 mL of 1 M HCl was added, forming a white precipitate. This solution was stirred and heated at 45–50 °C until flocculation, and then filtered using minimal water. Last, addition of 2.0 mL of concentrated HCl to the filtrate resulted in the formation of cisplatin, which was refrigerated overnight before filtering and washing with cold ethanol and cold 0.1 M HCl. The product was dried in a vacuum oven for 2 h, resulting in 0.27 g of yellow-orange cisplatin (90% yield). Molecular weight (301.1 g/mol), and expected peak pattern, were confirmed by mass spectrometry (Supplementary Material). RP-HPLC (C-18, 1 mL/min, 0.1% TFA/water to 40% 0.1%TFA/acetonitrile over 20 min) at 305 nm showed a single peak at 5.48 min (purity > 99%); see Supplementary material. This was consistent with commercial cisplatin purchased previously from Sigma-Aldrich (PHR1624, lot LRAB7778), which gave a single peak at 5.46 min under the same conditions.

### 4.6. Activated EGFR Assay

Activated (phosphorylated) EGFR was quantitated using the Phospho-EGFR (Y1173) + Total In-Cell ELISA Kit (ab207463) from Abcam (Waltham, MA USA), according to the manufacturer’s recommendation. Briefly, cells were cultured in 96-well plates and treated as indicated. The cells were then rapidly fixed, followed by incubation of each well with a primary antibody that recognized either phospho-EGFR (Tyr1173) or total-EGFR. The signal was then quantitated following incubation with a secondary HRP-conjugated antibody and a developing solution. The relative number of cells in each well was determined using the provided Crystal Violet solution. The phospho-EGFR and total- EGFR signals were then normalized to cell number, and the ratio of phosphorylated EGFR to total EGFR for each condition was calculated and plotted.

### 4.7. Activated IGF-1R Assay

Activated (phosphorylated) IGF-1R was quantitated using the Phospho-IGF-1R (Tyr1165/1166) and Total IGF-1R ELISA kit (RayBiotech, Norcross, GA, USA), according to the manufacturer’s instructions. Briefly, the same concentration of cell lysates, treated as indicated, was added to wells of a 96-well plate precoated with an anti-pan IGF-1R antibody that bound itself to the IGF-1R present in the sample. The wells were then washed and rabbit anti-phospho-IGF-1R (Tyr1165/1166) antibody was added to certain wells to detect phosphorylated IGF-1R, while biotinylated anti-pan-IGF-1R antibody was added to other wells to detect pan IGF-1R. After washing the wells, HRP-conjugated anti-rabbit IgG or HRP Streptavidin was added, along with a TMB substrate solution. Color development was measured in the linear range and in proportion to the amount of IGF-1R (Tyr1165/1166) or pan bound IGF-1R. Following addition of the stop solution, the color changed from blue to yellow, and then the intensity of the color was measured at 450 nm.

### 4.8. PI3K Assay

Activated phosphorylated-PI3K p85 + total PI3K p85 in-cell ELISA kit (Abcam) was used according to the recommendations by the manufacturer as we recently reported [40,76]. Briefly, cells were cultured in 96-well plates then treated as indicated. Following treatment, the cells were fixed, and the wells were then incubated with a primary antibody targeting either total PI3K p85 (recognizes the total level of PI3K p85 proteins regardless of the phosphorylation state) or phosphorylated-PI3K p85 (recognizes p85 PI3K alpha/gamma phospho-tyrosine 467/199). Secondary HRP-conjugated antibodies were then added, and the signal detected after addition of the developing solution. Crystal Violet solution was then added to determine the relative number of cells in each well. Signals for phospho-PI3K and total-PI3K were normalized to cell number then the ratio of phospho-PI3K to total-PI3K for each treatment was determined and plotted. 

### 4.9. AKT Assay

The AKT kinase activity assay kit (Abcam) was used to quantitate the activity of AKT according to the manufacturer’s instructions as we previously reported [40,76]. In brief, the assay is based on a solid phase ELISA. A specific synthetic peptide is used as a substrate for AKT along with a polyclonal antibody that binds the phosphorylated substrate. 

### 4.10. Statistical Analysis

The analysis was carried out as we previously reported [72,73,74,76]. Each experiment in this study was performed in triplicate and repeated a minimum of three times. Statistical values are expressed as the mean ± Standard Deviation (SD). To evaluate the statistical differences, the Mann–Whitney test was performed. All the statistical tests were two-sided and a *p* value of <0.05 was considered statistically significant in all cases. GraphPad Prism (GraphPad Software, 9.4.1) was used for the statistical analysis.

## Data Availability

Not applicable.

## Supplementary Materials

The supporting information can be downloaded at: https://www.mdpi.com/article/10.3390/ijms232112829/s1.
