# Supplementary material for: Regulation of Cisplatin Resistance in Lung Cancer Cells by Nicotine, BDNF, and a β-Adrenergic Receptor Blocker
Source: Int J Mol Sci. 2022 Oct 24;23(21):12829. doi: 10.3390/ijms232112829 (PMC9657603; doi:10.3390/ijms232112829)
Supplement: Supplementary file 1 [file ijms-23-12829-s001.zip › ijms-1924568-SI.pdf]

Supplementary Data

Manuscript ID: ijms-1924568

Title: Regulation of Cisplatin Resistance in Lung Cancer Cells by Nicotine, BDNF, and a β-Adrenergic Receptor Blocker

Authors: Ravel Ray, Hind Al Khashali, Ben Haddad, Jadziah Wareham, Kai-Ling Coleman, Danyah Alomari, Robert Ranzenberger, Jeffrey Guthrie, Deborah Heyl, Hedeel Guy Evans*

Cisplatin synthesized in this study

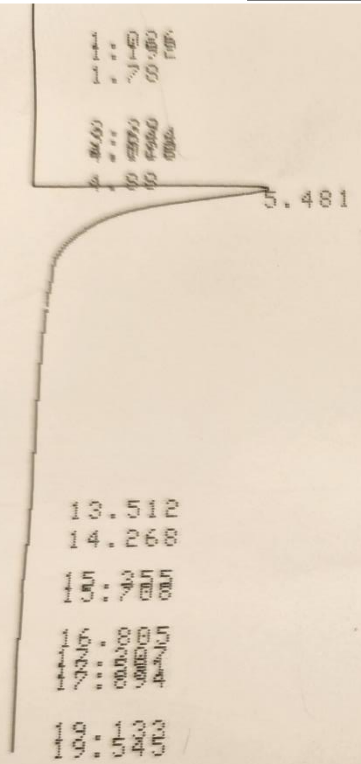

CHROMATOGRAM 1 MEMORIZED

CR501 CHROMATOPAC
CHANNEL NO 1
SAMPLE NO 0
REPORT NO 17

FILE 0
METHOD 41

PKNO	TIME	AREA	MK	IDNO	CONC	NAME
1	5.481	3902510	SV		100	
	TOTAL	3902510			100	

Theoretical isotope pattern for cisplatin

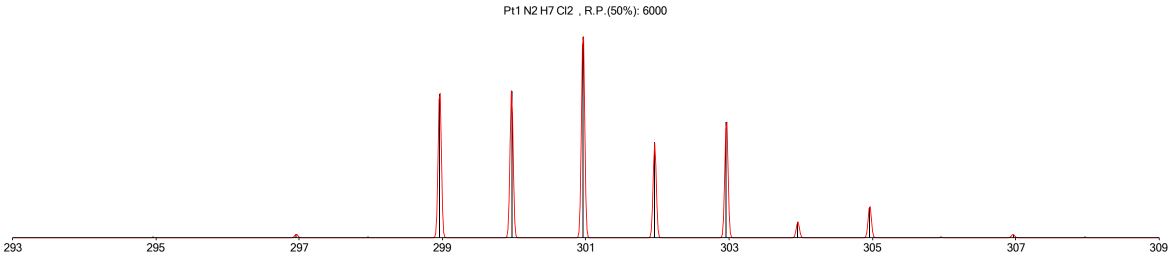

Calculated Isotope Distribution

Composition: Pt1 N2 H7 Cl2

Unsaturation: -2.5

m/z	Rel. Abundance	
294.95856	0.0300	
296.95962	1.6950*	
297.95756	0.0140	
298.96122	71.7080	************************************
299.96337	73.0750	*************************************
300.96118	100.0000	**
301.96043	47.1510	************************
302.96152	57.5150	*****************************
303.95762	7.8740****	
304.96141	15.3970	********
305.95937	0.1260	
306.96060	1.5680*	
307.95855	0.0130	

Calculated average molecular weight: 301.05194
Percent composition:
Pt: 64.799% N: 9.305% H: 2.344% Cl: 23.552%

Cisplatin

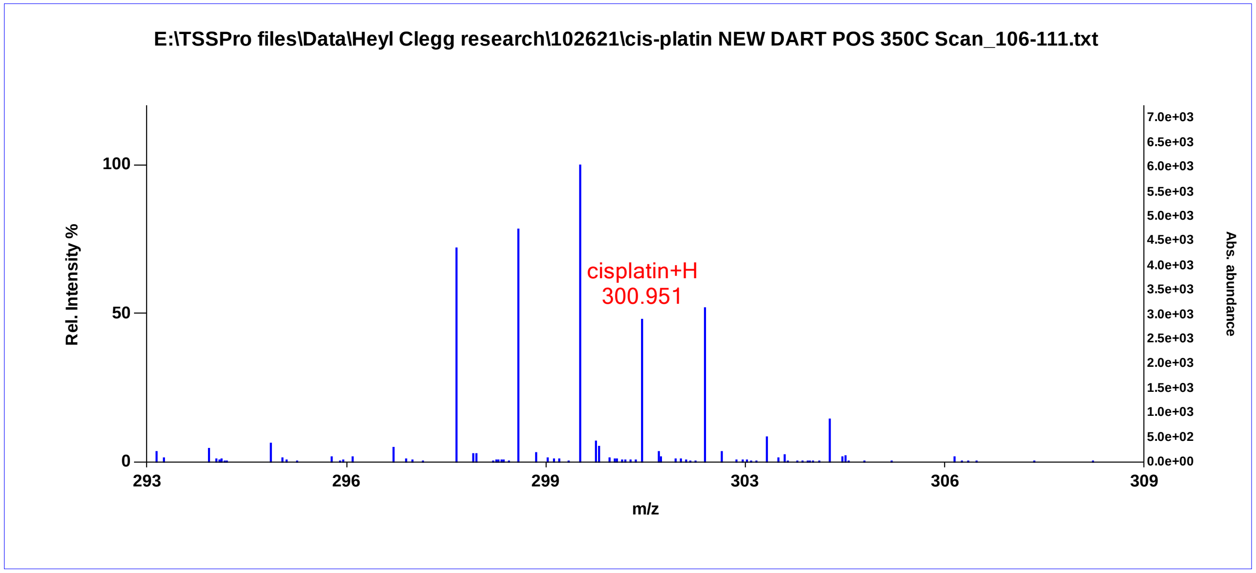

E:\TSSPro files\Data\Heyl Clegg research\102621\cis-platin NEW DART POS 350C Scan_106-111.txt
Rel. Intensity %
100
50
0
293
296
299
303
306
309
m/z
cisplatin+H
300.951
7.0e+03
6.5e+03
6.0e+03
5.5e+03
5.0e+03
4.5e+03
4.0e+03
3.5e+03
3.0e+03
2.5e+03
2.0e+03
1.5e+03
1.0e+03
5.0e+02
0.0e+00
Abs. abundance

Cisplatin purchased from Sigma Aldrich

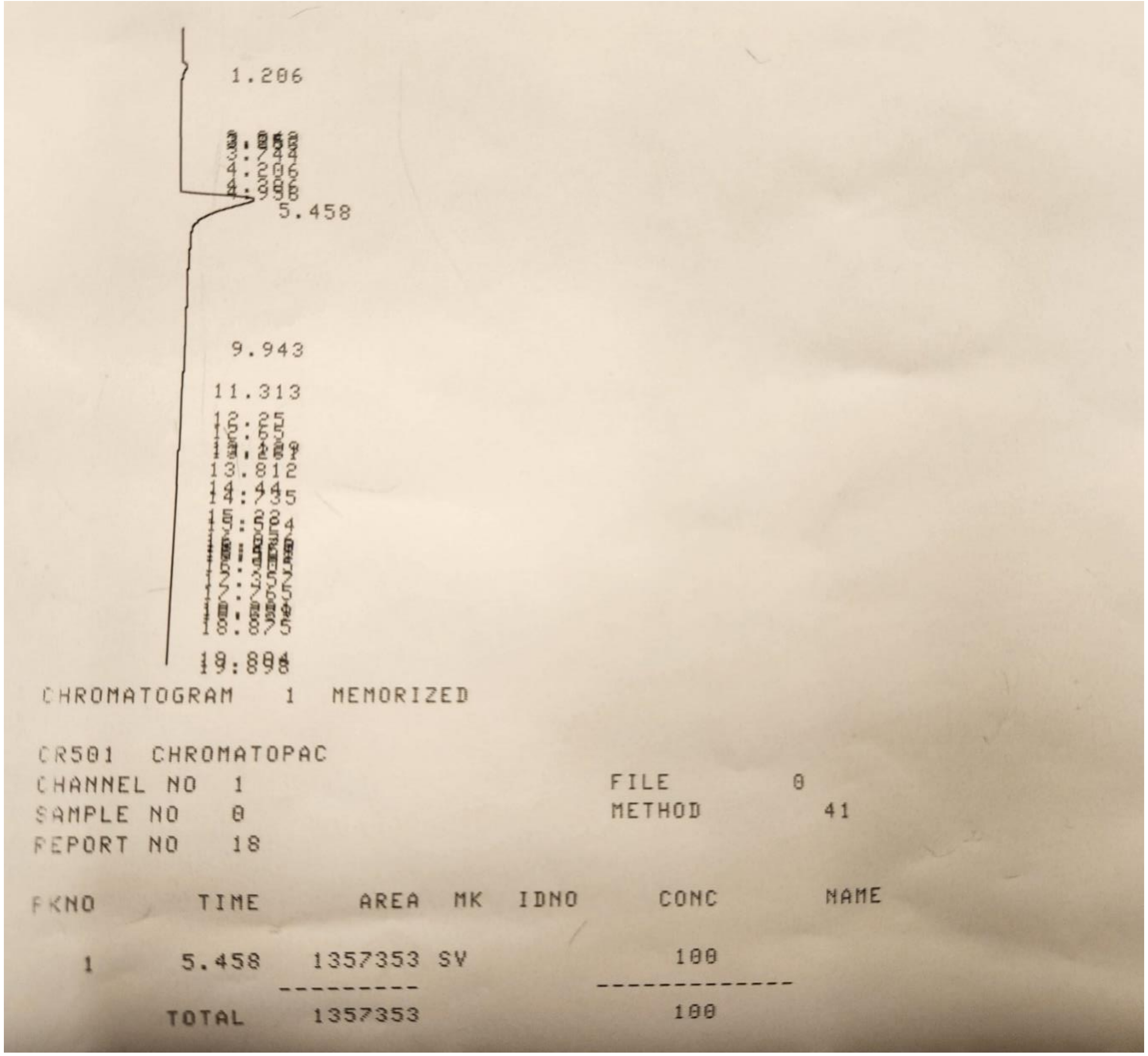

CHROMATOGRAM 1 MEMORIZED

CR501 CHROMATOPAC

CHANNEL NO 1 FILE 0

SAMPLE NO 0 METHOD 41

REPORT NO 18

PKNO	TIME	AREA	MK	IDNO	CONC	NAME
1	5.458	1357353	SV		100	
	TOTAL	1357353			100	

Cell viability of 24h and 48h treatments

Cisplatin Concentration (μM)	(A549) 48 hours			AVE	(A549 + Propranolol) 48 hours			AVE	(A549 + BDNF) 48 hours			AVE	(A549 + Nicotine) 48 hours			AVE
0	93.86598	100.2062	105.9278	100	93.86598	100.2062	105.9278	100	104.8358	101.1405	94.02372	100	103.2809	99.64045	96.90337	99.94157
2.5	92.78351	77.31959	85.05155	85.05155	77.31959	65.72165	74.69072	72.57732	88.95985	95.80292	104.0146	96.25912	103.1461	94.38202	102.4719	100
5	77.31959	61.85567	75	71.39175	42.33247	44.84536	57.75541	48.31108	98.54015	102.646	87.59124	96.25912	94.38202	101.1236	86.29213	93.93258
7.5	61.85567	54.12371	50.25773	55.41237	35.56701	33.44613	49.09794	39.37036	88.95985	94.43431	78.14781	87.18066	95.25843	80.89888	76.98876	84.38202
10	54.12371	46.00515	65.33505	55.15464	33.76237	46.00515	48.28608	42.68454	79.65328	76.7792	82.66423	79.69891	95.08315	82.24719	81.4382	86.25618
15	46.39175	65.33505	58.10567	56.61082	31.97165	18.9433	19.44588	23.45361	62.9562	67.13047	62.9562	64.34763	80.89888	68.08989	80.89888	76.62921
20	46.39175	30.92784	40.97938	39.43299	19.3299	10.82474	25.51546	18.5567	47.90146	56.93431	59.26095	54.69891	81.50562	73.02472	71.86517	75.46517
30	40.14433	23.19588	31.77526	31.70515	8.443299	17.78351	24.0433	16.7567	43.68613	44.4115	35.99453	41.36405	67.85958	55.88764	61.21838	61.6552
40	15.7732	24.12371	31.76675	23.88789	16.25289	9.278351	8.358905	11.29671	38.32117	50.91241	35.0365	41.42336	57.70787	45.46517	43.90112	49.02472
60	10.20619	18.40206	9.981959	12.8634	24.21649	9.572165	9.981959	14.59021	30.93066	17.65511	29.05566	25.88047	30.47191	30.8764	42.10787	34.48539
80	7.289459	8.079124	8.041237	7.803273	8.062655	7.843995	7.268041	7.724897	14.89051	24.90876	20.65374	20.151	14.66966	24.53933	28.19326	22.46742

Cisplatin Concentration (μM)	(A549) 24 hours			AVE	(A549 + Propranolol) 24 hours			AVE	(A549 + BDNF) 24 hours			AVE	(A549 + Nicotine) 24 hours			AVE
0	94.03801	100.755	105.207	100	99.60464	98.00963	102.3857	100	103.3155	101.4716	95.14155	99.97622	101.6913	99.09472	97.06779	99.28461
2.5	96.06873	85.91513	89.82036	90.60141	82.99507	81.48606	90.99277	85.15797	99.12962	95.15905	100.2737	98.18745	101.1482	100.0221	98.82976	100
5	93.72559	84.35303	85.91513	87.99792	63.94393	48.89164	63.80812	58.88123	99.86989	101.014	99.11616	100	98.5648	100.0221	91.86134	96.81607
7.5	76.54257	79.66675	67.56053	74.58995	41.19573	37.21725	50.92879	43.11392	95.09175	94.98273	90.5828	93.55242	98.121	92.60322	80.42835	90.38419
10	60.14059	67.21648	73.8089	67.05532	37.47303	43.72331	47.26946	42.82193	91.2827	88.30813	84.84903	88.14662	96.07419	91.29168	88.81431	92.06006
15	59.35954	62.8352	66.50612	62.90029	33.4621	26.03027	21.99369	27.16202	74.64669	73.74557	67.24393	71.87873	92.60322	75.5796	85.44933	84.54405
20	47.64384	57.79745	43.19188	49.54439	24.52127	16.70464	25.87937	22.36843	72.00861	63.47526	66.26542	67.24976	83.54824	79.41038	77.41179	80.12347
30	41.54543	34.60036	36.79479	37.64686	14.57695	19.84337	24.97095	19.79709	56.95976	58.16479	42.57257	52.56571	71.42239	62.86156	66.17885	66.82093
40	32.88519	30.55454	24.27883	29.23952	15.8599	9.054007	8.156793	11.02357	45.73556	54.31199	45.17026	48.40594	62.63634	57.39015	51.99823	57.34158
60	24.46238	18.58891	16.62926	19.89352	17.59495	10.74258	9.800963	12.71283	36.69074	18.0358	41.33712	32.02122	36.56436	37.47847	44.14617	39.39633
80	16.35728	8.236058	9.403801	11.33238	7.868687	7.652824	8.242165	7.921225	17.10548	26.89488	27.12182	23.70739	27.66174	25.06514	30.61724	27.78137

Cell viability of 24h and 48h treatments

Cisplatin Concentration (µM)	(H1299) 48 hours			AVE	(H1299 + Propranolol) 48 hours			AVE	(H1299 + BDNF) 48 hours			AVE	(H1299 + Nicotine) 48 hours			AVE
0	92.22248	102.3309	95.13039	96.56127	95.50669	105.9751	98.51816	100	98.15703	103.7617	98.08129	100	93.91304	99.27536	93.84058	95.67633
2.5	97.8306	96.93053	105.2389	100	92.71033	77.00765	78.52772	82.74857	98.45998	102.3226	90.88614	97.22292	94.2029	97.89855	102.8986	98.33333
5	96.93053	103.8541	98.10755	99.63074	62.6673	65.24857	57.36138	61.75908	98.45998	94.67306	99.97475	97.7026	94.2029	105.0725	95.65217	98.30918
7.5	90.00692	94.16109	98.31526	94.16109	62.5956	51.62524	67.54302	60.58795	90.88614	102.2469	96.18783	96.44029	110.1449	97.82609	92.02899	100
10	97.78906	105.3773	85.02192	96.06277	62.55258	62.75478	51.826	59.04446	96.94522	98.45998	93.91568	96.44029	92.75362	103.6232	89.85507	95.41063
15	91.39165	76.1597	75.60582	81.05239	51.62524	42.37572	57.79159	50.59751	92.40091	98.45998	90.88614	93.91568	88.4058	94.2029	110.1449	97.58454
20	76.1597	82.27464	69.2361	75.89015	36.56788	45.05163	35.85086	39.15679	90.88614	98.45998	87.09922	92.14845	86.95652	94.2029	102.5362	94.56522
30	54.2811	47.77291	59.03069	53.6949	35.73614	27.89197	37.71511	33.78107	71.95153	90.88614	75.73845	79.52537	92.02899	85.50725	95.28986	90.94203
40	38.77221	43.92338	29.8546	37.51673	18.35564	15.37285	22.37094	18.69981	75.73845	53.01691	60.59076	63.11537	86.95652	79.71014	81.15942	82.6087
60	31.29471	17.86291	29.39765	26.18509	15.07203	18.49904	17.63862	17.0699	32.09796	32.78793	40.35875	35.08155	38.98551	31.37029	44.87889	38.41156
80	15.06577	25.20194	15.10732	18.45834	1.560229	14.36644	13.04971	9.658795	7.913911	18.17723	15.49609	13.86241	22.06449	17.3913	23.22174	20.89251

Cisplatin Concentration (µM)	(H1299) 24 hours			AVE	(H1299 + Propranolol) 24 hours			AVE	(H1299 + BDNF) 24 hours			AVE	(H1299 + Nicotine) 24 hours			AVE
0	94.52291	101.6741	94.61936	96.9388	98.26231	102.768	98.96972	100	98.24743	101.7526	98.09214	99.36405	95.00948	101.1375	95.00875	97.05192
2.5	97.91939	99.3455	102.7351	100	97.01978	98.09126	92.83883	95.98329	98.5802	101.8642	97.82741	99.42394	95.52282	99.54062	97.94371	97.66905
5	97.82983	101.2745	97.94695	99.01711	82.66545	75.78964	89.00068	82.48526	101.0131	96.09554	100.8223	99.31031	98.00204	98.07496	102.0271	99.36804
7.5	88.80468	92.01516	100.7234	93.84774	89.90459	75.70064	74.34477	79.98333	98.66154	103.4903	97.81114	99.98768	101.7355	98.43955	96.76243	98.97915
10	99.23527	104.1819	91.13331	98.18349	75.55601	74.76196	61.69054	70.6695	97.07905	100.1405	97.77712	98.33222	96.83535	100.9406	96.54368	98.10656
15	93.14502	90.25146	89.01137	90.80262	64.46986	55.0205	61.88745	60.45927	100.7321	101.405	97.8629	100	100.7875	100.6271	98.58539	100
20	81.98415	90.13572	89.56252	87.22747	47.63622	57.59456	46.23863	50.48981	98.88338	99.54152	90.32759	96.25083	90.71022	95.51553	104.4298	96.88518
30	75.67344	61.1092	68.38443	68.38902	34.65464	27.04786	36.57372	32.75874	95.39303	81.55513	86.96295	87.97037	92.36547	87.63308	98.01298	92.67051
40	45.19463	50.5298	44.33483	46.68642	29.66226	16.97577	28.02965	24.88922	83.11765	68.91962	82.15633	78.06453	90.71022	90.35292	80.35015	87.13776
60	41.23458	27.41991	33.96486	34.20645	14.61589	19.87218	17.10482	17.19763	46.76329	36.41137	39.40472	40.85979	56.52166	69.20665	55.55876	60.42902
80	16.65587	16.2866	31.56734	21.50327	15.15515	13.93167	15.25666	14.78116	12.60438	21.01827	15.11203	16.24489	24.02581	39.83083	23.65174	29.16946

Activated EGFR assay

Activated (phosphorylated) EGFR was quantitated using the Phospho-EGFR (Y1173) + Total In-Cell ELISA Kit (ab207463) from Abcam according to the manufacturer's recommendation.

The number of cells in each well was determined using the provided Crystal Violet solution in the kit. The phospho-EGFR and total- EGFR ODs at 450 nm were then normalized to cell number for each well/condition, then the ratio of phosphorylated EGFR to total EGFR for each condition was calculated as described in the Methods section and Figure 3 legend. The data was normalized to the control of each cell line. Shown below is one data set out of a total of five independent assays used for Figure 3.

	A549 Phospho-EGFR (Y1173)					A549 Total					A549	A549
	1	2	3	AVG	SD	1	2	3	AVG	SD	Phos/Total	Norm to Con
Control	0.287	0.307	0.304	0.299333	0.010786	1.266	1.245	1.238	1.249667	0.014572	0.23953054	1
Epinephrine (100nM)	0.425	0.481	0.442	0.449333	0.028711	1.348	1.325	1.319	1.330667	0.015308	0.33767535	1.40973818
Propranolol (1µM)	0.112	0.201	0.11	0.141	0.051971	1.263	1.245	1.251	1.253	0.009165	0.11252993	0.46979365
Epinephrine (100nM) + Propranolol (1µM)	0.318	0.255	0.205	0.259333	0.056624	1.347	1.331	1.329	1.335667	0.009866	0.19416022	0.81058648
Nicotine (1 µM)	0.618	0.455	0.698	0.590333	0.12384	1.322	1.319	1.358	1.333	0.021703	0.44286072	1.84886951
Nictine (1µM) + Propranolol (1 µM)	0.337	0.417	0.413	0.389	0.045078	1.428	1.397	1.388	1.404333	0.020984	0.27699976	1.15642774
BDNF (5nM)	0.342	0.348	0.362	0.350667	0.010263	1.292	1.334	1.387	1.337667	0.047606	0.26214802	1.09442419
BDNF (5nM) + nicotine (1µM)	0.665	0.612	0.597	0.624667	0.035726	1.313	1.32	1.367	1.333333	0.029366	0.4685	1.95590924
BDNF (5nM) + propranolol (1µM)	0.202	0.168	0.186	0.185333	0.01701	1.282	1.301	1.298	1.293667	0.010214	0.14326205	0.59809511
BDNF (5nM) + nic (1µM) + prop (1µM)	0.461	0.503	0.437	0.467	0.033407	1.287	1.265	1.301	1.284333	0.018148	0.36361277	1.51802257
Erlotinib (10 µM)	0.028	0.048	0.044	0.04	0.010583	1.396	1.313	1.301	1.336667	0.051733	0.02992519	0.12493266
Erlotinib (10 µM) + BDNF (5 nM)	0.036	0.038	0.038	0.037333	0.001155	1.172	1.265	1.301	1.246	0.066566	0.02996255	0.12508863
Erlotinib (10 µM) + nicotine (1µM)	0.046	0.024	0.034	0.034667	0.011015	1.169	1.206	1.301	1.225333	0.068091	0.02829162	0.11811279
Erlotinib (10 µM) + propranolol (1µM)	0.0306	0.047	0.04	0.0392	0.008229	1.293	1.332	1.356	1.327	0.031796	0.02954032	0.12332589

	H1299 Phospho-EGFR (Y1173)					H1299 Total					H1299	H1299
	1	2	3	AVG	SD	1	2	3	AVG	SD	Phos/Total	Norm to Con
Control	0.242	0.331	0.353	0.308667	0.078489	1.477	1.44	1.411	1.442667	0.04666905	0.21395564	1
Epinephrine (100nM)	0.628	0.562	0.475	0.555	0.108187	1.473	1.431	1.402	1.435333	0.05020458	0.38666976	1.80724269
Propranolol (1µM)	0.1566	0.146	0.161	0.154533	0.003111	1.273	1.335	1.402	1.336667	0.09121677	0.11561097	0.5403502
Epinephrine (100nM) + Propranolol (1µM)	0.287	0.237	0.232	0.252	0.038891	1.426	1.389	1.402	1.405667	0.01697056	0.17927437	0.83790438
Nicotine (1 µM)	0.635	0.627	0.644	0.635333	0.006364	1.395	1.404	1.355	1.384667	0.02828427	0.45883486	2.14453268
Nictine (1µM) + Propranolol (1 µM)	0.402	0.366	0.412	0.393333	0.007071	1.364	1.333	1.321	1.339333	0.03040559	0.29367845	1.37261373
BDNF (5nM)	0.314	0.291	0.311	0.305333	0.002121	1.229	1.345	1.302	1.292	0.0516188	0.23632611	1.10455659
BDNF (5nM) + nicotine (1µM)	0.698	0.617	0.703	0.672667	0.003536	1.407	1.412	1.399	1.406	0.00565685	0.47842579	2.2360981
BDNF (5nM) + propranolol (1µM)	0.145	0.118	0.245	0.169333	0.070711	1.355	1.303	1.345	1.334333	0.00707107	0.12690482	0.59313614
BDNF (5nM) + nic (1µM) + prop (1µM)	0.483	0.577	0.567	0.542333	0.059397	1.423	1.356	1.347	1.375333	0.05374012	0.39432865	1.84303929
Erlotinib (10 µM)	0.021	0.028	0.056	0.035	0.024749	1.379	1.355	1.302	1.345333	0.05444722	0.02601586	0.12159463
Erlotinib (10 µM) + BDNF (5 nM)	0.031	0.031	0.044	0.035333	0.009192	1.361	1.304	1.378	1.347667	0.01202082	0.02621815	0.12254014
Erlotinib (10 µM) + nicotine (1µM)	0.035	0.026	0.044	0.035	0.006364	1.321	1.305	1.354	1.326667	0.02333452	0.02638191	0.12330551
Erlotinib (10 µM) + propranolol (1µM)	0.023	0.041	0.042	0.035333	0.013435	1.3549	1.304	1.321	1.326633	0.02397092	0.02663384	0.12448298

Note: After we collected the data for this manuscript, the kit became unavailable. The company communicated to us that the reason was not at all due to any problems of any sort with the kit but rather the unavailability at this time was due to commercial reasons. From the manufacturer: *"There has been no quality issue and you can use the remaining stock that you have without any considerations."*
